# Neuropathic pain: an updated grading system for research and clinical practice

**DOI:** 10.1097/j.pain.0000000000000492

**Published:** 2016-01-13

**Authors:** Nanna B. Finnerup, Simon Haroutounian, Peter Kamerman, Ralf Baron, David L.H. Bennett, Didier Bouhassira, Giorgio Cruccu, Roy Freeman, Per Hansson, Turo Nurmikko, Srinivasa N. Raja, Andrew S.C. Rice, Jordi Serra, Blair H. Smith, Rolf-Detlef Treede, Troels S. Jensen

**Affiliations:** a Danish Pain Research Center, Department of Clinical Medicine, Aarhus University, Aarhus, Denmark; b Division of Clinical and Translational Research, Department of Anesthesiology, Washington University School of Medicine, St. Louis, MO, USA; c Brain Function Research Group, School of Physiology, Faculty of Health Sciences, University of the Witwatersrand, Johannesburg, South Africa; d Division of Neurological Pain Research and Therapy, Department of Neurology, Universitätsklinikum Schleswig-Holstein, Campus Kiel, Kiel, Germany; e Nuffield Department of Clinical Neuroscience, University of Oxford, Oxford, United Kingdom; f INSERM U-987, Centre d'Evaluation et de Traitement de la Douleur, CHU Ambroise Paré, Boulogne-Billancourt, France; g Université Versailles-Saint-Quentin, Versailles, France; h Department of Neurology and Psychiatry, Sapienza University, Rome, Italy; i Autonomic and Peripheral Nerve Laboratory, Department of Neurology, Beth Israel Deaconess Medical Center, Harvard Medical School, Boston, MA, USA; j Department of Pain Management and Research, Division of Emergencies and Critical Care, Oslo University Hospital, Oslo, Norway; k Department of Molecular Medicine and Surgery, Karolinska Institutet, Stockholm, Sweden; l Pain Research Institute, Neuroscience Research Centre, The Walton Centre NHS Foundation Trust, Liverpool, United Kingdom; m Division of Pain Medicine, Department of Anesthesiology and Critical Care Medicine, Johns Hopkins School of Medicine, Baltimore, MD, USA; n Pain Research, Department of Surgery and Cancer, Faculty of Medicine, Imperial College London, United Kingdom; o Pain Medicine, Chelsea and Westminster Hospital NHS Foundation Trust, London, United Kingdom; p Neuroscience Technologies, Ltd, Barcelona, Spain; q Ninewells Hospital and Medical School, Division of Population Health Sciences, School of Medicine, University of Dundee, Dundee, Scotland; r Chair of Neurophysiology, Center of Biomedicine and Medical Technology Mannheim, Medical Faculty Mannheim, Heidelberg University, Germany; s Department of Neurology, Aarhus University Hospital, Aarhus, Denmark

**Keywords:** Neuropathic pain, Definition, Grading, Possible, Probable, Definite

## Abstract

The redefinition of neuropathic pain as “pain arising as a direct consequence of a lesion or disease affecting the somatosensory system,” which was suggested by the International Association for the Study of Pain (IASP) Special Interest Group on Neuropathic Pain (NeuPSIG) in 2008, has been widely accepted. In contrast, the proposed grading system of possible, probable, and definite neuropathic pain from 2008 has been used to a lesser extent. Here, we report a citation analysis of the original NeuPSIG grading paper of 2008, followed by an analysis of its use by an expert panel and recommendations for an improved grading system. As of February, 2015, 608 eligible articles in Scopus cited the paper, 414 of which cited the neuropathic pain definition. Of 220 clinical studies citing the paper, 56 had used the grading system. The percentage using the grading system increased from 5% in 2009 to 30% in 2014. Obstacles to a wider use of the grading system were identified, including (1) questions about the relative significance of confirmatory tests, (2) the role of screening tools, and (3) uncertainties about what is considered a neuroanatomically plausible pain distribution. Here, we present a revised grading system with an adjusted order, better reflecting clinical practice, improvements in the specifications, and a word of caution that even the “definite” level of neuropathic pain does not always indicate causality. In addition, we add a table illustrating the area of pain and sensory abnormalities in common neuropathic pain conditions and propose areas for further research.

## 1. Introduction

In 1994, the International Association for the Study of Pain (IASP) defined neuropathic pain as “pain initiated or caused by a primary lesion or dysfunction in the nervous system.” In 2008, a task force initiated by the IASP Special Interest Group on Neuropathic Pain (NeuPSIG) noted the need to distinguish neuropathic pain from nociceptive pain arising indirectly from neurological disorders and pain conditions with secondary neuroplastic changes occurring in the nociceptive system, and proposed a new definition that omitted the term “dysfunction”: “pain arising as a direct consequence of a lesion or disease affecting the somatosensory system.”^30^ A slightly modified version of this definition was proposed by the IASP Taxonomy Committee and accepted by the IASP: “pain caused by a lesion or disease of the somatosensory nervous system.”^16,17^ The omission of the term “dysfunction” excludes conditions involving ill-defined changes in the nervous system and conditions with no known lesion of the somatosensory nervous system from being classified as neuropathic pain. The restriction to the somatosensory nervous system is important because conditions such as musculoskeletal pain (eg, due to spasticity) arising indirectly from disorders of the motor system should not be confused with neuropathic pain. The term “primary” was omitted because of the difficulty in distinguishing between primary and secondary causes of neuropathic pain; however, the omission means that nociceptive pain conditions that—over time—may cause secondary lesions in the somatosensory nervous system could ultimately be considered as being partly neuropathic pain.

Recognizing the challenges of determining the presence of neuropathic pain according to this new definition, NeuPSIG also proposed a grading system^30^ to guide decisions on the level of certainty with which neuropathic pain can be determined in an individual patient. Three levels of certainty—possible, probable, and definite neuropathic pain—were proposed. As an activity in the Global Year Against Neuropathic Pain,^15^ NeuPSIG established a committee to (1) critically evaluate the use of the grading system in the 7 years after its publication, (2) assess the usefulness and limitations of the grading system, and (3) update the grading system if required, for improved application in clinical and research settings. The committee consisted of an expert panel of neurologists, clinical neurophysiologists, neuroscientists, anesthesiologists, pain specialists, primary care physicians, and population health scientists.

## 2. Procedure

To generate background material and discussion points, we performed a systematic literature search using the Scopus database, which is an abstract and citation database of peer-reviewed literature (including scientific journals, books, and conference proceedings). This database was searched on February 6, 2015 for publications that cited the original NeuPSIG grading paper from 2008.^30^ Three review authors (S.H., P.K., and N.B.F.) extracted the following data: (1) use of the citation, (2) classification of the publication as a review, animal or human experimental paper, a clinical study, or others, (3) criteria used for including or classifying patients with neuropathic pain in clinical studies, (4) comparison of the grading with other criteria for identifying neuropathic pain when available, and (5) any issues raised with the grading system.

In addition, all committee members were asked to examine the grading system for possible deficiencies that could require modification or amendment in a subsequent iteration of the grading system. Participants convened under the auspices of NeuPSIG in Nice, France, on May 14, 2015. Before the meeting, all participants were provided with a documentation folder that included results from the literature review and issues identified by committee members. At the meeting, data on the use of the grading paper were presented, and individual participants provided short overviews on issues with the grading system that had been identified before the meeting. Discussions pertaining to the issues identified before the meeting and new issues raised by participants at the meeting were used to inform modifications to the existing grading system. Before and after the meeting, the process and update were discussed through e-mail, and after circulating draft manuscripts through e-mail, a final update was agreed through consensus by e-mail.

## 3. Background material

A total of 731 publications were identified in Scopus as citing the original grading paper^30^ at the time of search, which represented about 5% of all publications in Scopus with the term “neuropathic pain” in the title, abstract, or keywords in the same period. Of the 731 publications, 123 were not available as full-text at any of the 3 institutions, at which the reviewers were based or were in a language not understood by the reviewers. Hence, the full text of 608 publications was downloaded and used to evaluate the use of the original grading paper since its publication in 2008. Of the 608 included publications, 269 were classified by the reviewers as reviews or book chapters, 220 as clinical studies, 73 as experimental studies, and 46 as “others.”

**Figure 1.**
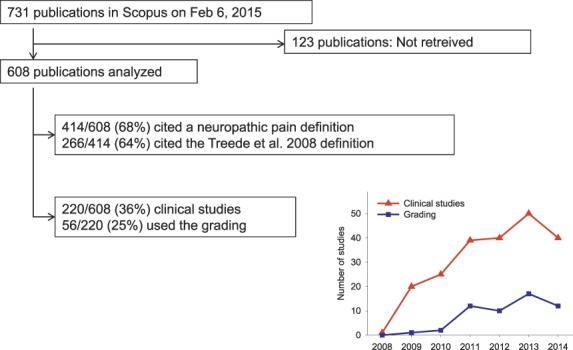
Summary of how the citations of the neuropathic pain grading paper^30^ were used. The figure indicates the percentage of 608 publications that cited the original grading paper^30^ for defining neuropathic pain and the number of clinical studies that used the grading system for identifying neuropathic pain. The insert indicates the total number of clinical studies and the number of studies using the grading system^30^ for identifying neuropathic pain per year.

Of the 608 publications, 414 cited the grading paper^30^ in relation to the definition of neuropathic pain (Fig. 1). Of these, 266 used the definition as it was presented (or very similar) in the original grading paper, whereas 48 applied the adapted 2011 IASP definition^17^ and 8 applied the 1991 IASP definition despite using the grading paper as the reference. Ninety-two presented other definitions of neuropathic pain, of which most had a wording consistent with the definition in the grading paper, whereas others presented a definition significantly different despite using the grading paper as reference. The grading paper was cited in relation to other statements, unrelated to the definition or grading system, in 190 publications.

Of the 220 clinical publications that included patients, only 56 (25% of clinical studies, 9% of all studies citing the grading paper) used the grading system to include or classify patients as having possible, probable, or definite neuropathic pain. A further 16 (7%) used other criteria for classification of pain, but retrospectively noted whether the patients had possible, probable, or definite neuropathic pain according to the grading system. The percentage of clinical studies citing the grading system that also used it to include or classify patients with neuropathic pain increased from 5% (1/20) in 2009 to 30% (12/40) in 2014 (Fig. 1). Of the remaining 148 studies that did not use the grading system for patient classification, 115 used other criteria to include or classify neuropathic pain patients. Of these, 50 used one or more questionnaires, 30 used Douleur Neuropathique en 4 questions, 11 used painDETECT, 8 LANSS (Leeds Assessment of Neuropathic Symptoms and Signs) or S-LANSS (self-report LANSS), 2 used McGill pain questionnaire, 1 used ID-Pain, 1 used standardized evaluation of pain, and 1 used the German pain questionnaire; 51 used various criteria including pain history, pain descriptors, clinical examination, and laboratory investigations; 2 used patient self-report; and 12 did not mention the criteria used.

Thus, the 2008 grading paper was mostly cited for the redefinition of neuropathic pain. The redefinition has since been introduced in the IASP terminology with minor modifications, and hence the authors' aim “to develop a more precise definition of neuropathic pain that will be useful for clinical and research purposes” has largely been achieved. The adoption of the grading system has naturally happened after a delay, and since 2011 a steady ratio of about 1/3 of clinical trials in the field have used it (Fig. 1).

A meta-analysis of cancer trials^4^ indicated that of the 4 criteria of the grading system, criterion 2 “a history suggestive of a relevant lesion or disease affecting the peripheral or central somatosensory system” and criterion 3 “demonstration of the distinct neuroanatomically plausible distribution by at least 1 confirmatory test” were available in the majority of trials (13-14 of 22), whereas criterion 1 “pain with a distinct neuroanatomically plausible distribution” was available less often (10/22) and confirmation of the underlying lesion of disease was rarely done (criterion 4, 7/22). This identifies 2 problems to be addressed in the present revision: (1) plausibility of pain distribution and its assessment and (2) need for establishing the neurological diagnosis by confirmatory tests.

In addition, the following deficiencies were identified from the paper reviews and discussed during the committee meeting: (1) Several screening tools (questionnaires) were developed before the redefinition of neuropathic pain by NeuPSIG and IASP^3,13^ but are not positioned in the grading system; (2) Some clinicians and investigators have difficulty in determining the topographical location of a lesion and its pathology, as the approach used in neurology of “where is the lesion?”, “what is the lesion?” is not intuitive to other medical disciplines; (3) Certain sensory signs are not specific to neuropathic pain; and (4) Determination of lesion type and location does not necessarily prove that the pain is caused by that lesion or disease (uncertainty of causal relationship).

**Figure 2.**
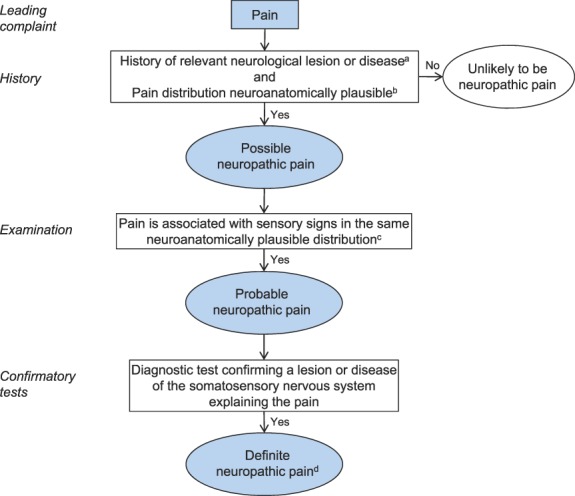
Flow chart of updated grading system for neuropathic pain. ^a^History, including pain descriptors, the presence of nonpainful sensory symptoms, and aggravating and alleviating factors, suggestive of pain being related to a neurological lesion and not other causes such as inflammation or non-neural tissue damage. The suspected lesion or disease is reported to be associated with neuropathic pain, including a temporal and spatial relationship representative of the condition; includes paroxysmal pain in trigeminal neuralgia. ^b^The pain distribution reported by the patient is consistent with the suspected lesion or disease (Table 1). ^c^The area of sensory changes may extend beyond, be within, or overlap with the area of pain. Sensory loss is generally required but touch-evoked or thermal allodynia may be the only finding at bedside examination. Trigger phenomena in trigeminal neuralgia may be counted as sensory signs. In some cases, sensory signs may be difficult to demonstrate although the nature of the lesion or disease is confirmed; for these cases the level “probable” continues to be appropriate, if a diagnostic test confirms the lesion or disease of the somatosensory nervous system. ^d^The term “definite” in this context means “probable neuropathic pain with confirmatory tests” because the location and nature of the lesion or disease have been confirmed to be able to explain the pain. “Definite” neuropathic pain is a pain that is fully compatible with neuropathic pain, but it does not necessarily establish causality.

Based on these limitations of the current grading system, we propose a change to the order of the grading criteria to better reflect clinical practice and have furthermore annotated the terms used to improve clarity (Fig. 2). In addition, a research agenda is proposed to further address shortcomings of the grading system.

## 4. Revised grading system

The grading system is intended for determining the level of certainty with which the pain in question is neuropathic. A finding of probable neuropathic pain in a given individual patient should prompt consideration of treatment according to the neuropathic pain treatment guidelines,^10^ but the grading system is not intended for medico-legal purposes or to classify diseases. The refinements in the present grading system (Fig. 2) follow the classical clinical method of diagnosis in that history, clinical examination, and diagnostic tests stepwise add to level of certainty that the pain in question is neuropathic.

### 4.1. Possible neuropathic pain

Evaluation of the patient according to the grading system should be undertaken if the patient's history suggests that pain could be related to a neurological lesion or disease and not other causes such as inflammation or non-neural tissue damage. At this stage, pain descriptors, the presence of nonpainful sensory symptoms, and any aggravating and alleviating factors can be taken into account. Pain descriptions such as burning or hot, electric shocks or shooting, pricking or pins and needles, pain evoked by light touching or cold, and nonpainful sensations such as numbness and tingling are suggestive, but not pathognomonic for neuropathic pain, and other descriptors may apply as well.^3^ The combination of several descriptors, however, has a highly discriminant value and several screening tools (questionnaires) have been developed to identify patients who may have neuropathic pain to alert the clinician to undertake further assessment (though they cannot be used alone to identify neuropathic pain).^3,13,32^ These include, but are not limited to the LANSS,^2^ the neuropathic pain questionnaire,^18^ the Douleur Neuropathique en 4 questions,^6^ the painDETECT,^11^ and ID-Pain.^24^

The following two criteria need to be fulfilled to reach the first level of certainty-“possible” neuropathic pain.

#### 4.1.1. A history of relevant neurological lesion or disease

There should be a clinical suspicion of a relevant lesion or disease of the somatosensory nervous system (eg, an episode of acute herpes zoster or a traumatic nerve injury). The temporal relationship between the lesion or disease and the pain may vary, but a close temporal relationship helps strengthen the clinical suspicion. The onset of pain is usually immediate or within a few weeks of the lesion or disease but may be delayed for up to several months after injury (eg, after stroke) or for many years in conditions with an insidious onset such as diabetic neuropathy. In some cases, the history of pain or sensory disturbances by themselves suggest a disease, eg, in polyneuropathy (peripheral neuropathy), where the insidious onset of distal pain or numbness may be the only history indicating the disease. Characteristic sudden short-lasting (usually a few seconds) paroxysmal pain in the face, which may recur several times and may be separated by a refractory period (usually some minutes), suggests trigeminal neuralgia, where the pain is the only symptom indicating a relevant neurological disease.

#### 4.1.2. Pain distribution neuroanatomically plausible

The pain distribution should be anatomically consistent with the suspected location of the lesion or disease in the peripheral or central somatosensory nervous system (as derived from the patient's history). This can be difficult to decipher in the single patient, as the distribution of pain can occupy a smaller area or extend somewhat outside the innervation territory of a peripheral nerve or root or the somatotopic representation of the body within the central nervous system, but it should be in a distribution that is typical for the underlying disorder (see examples in Table 1). In painful channelopathies, the pain distribution may be unusual but should be consistent with the disorder, eg, familial episodic pain syndrome, in which pain is mainly localized to the chest and upper arms, or inherited erythromelalgia, in which pain is localized to the extremities (feet and hands and in some cases ears).

**Table 1.**
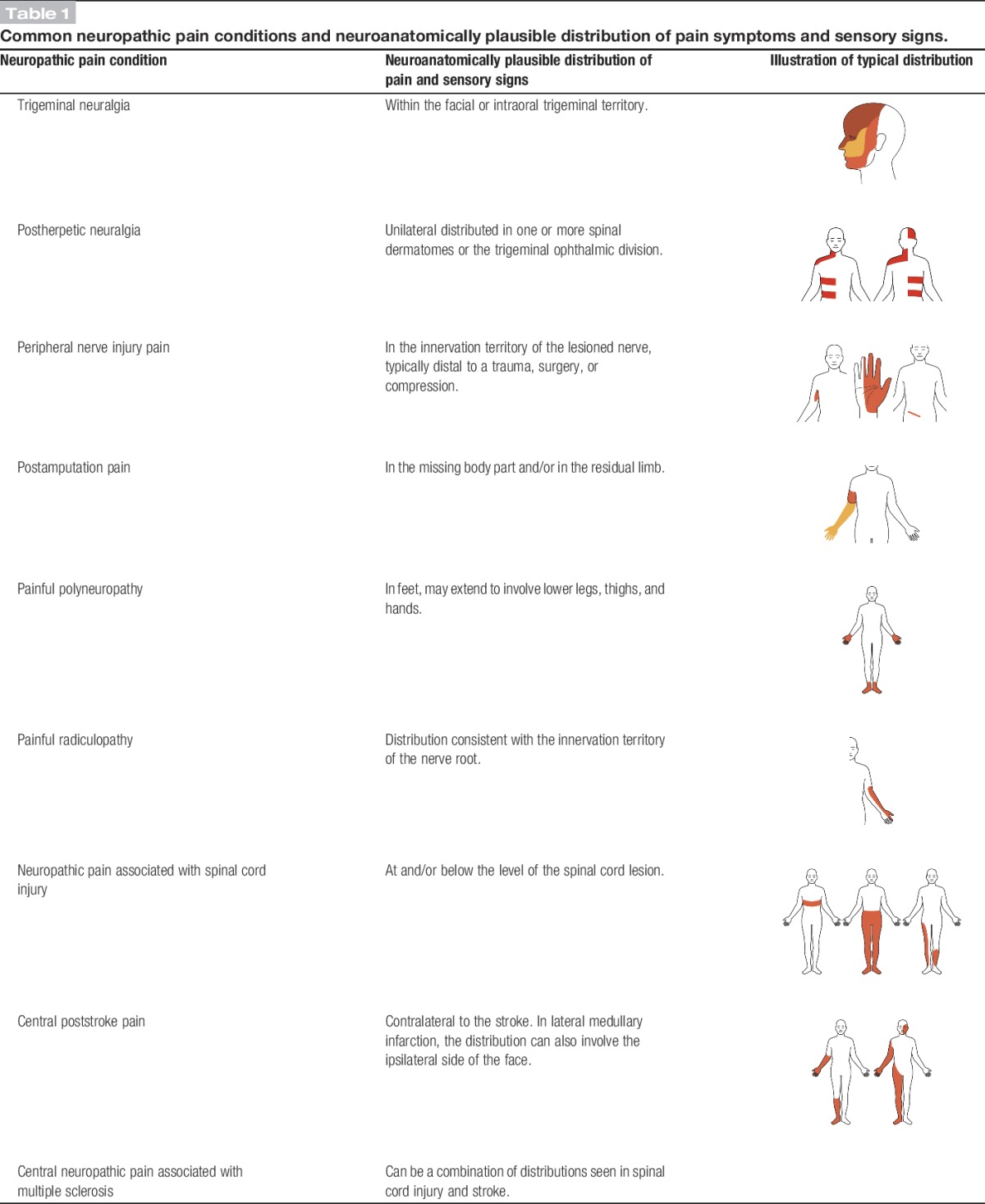
Common neuropathic pain conditions and neuroanatomically plausible distribution of pain symptoms and sensory signs.

When both requirements 1 and 2 of the pain history are fulfilled, the pain complaint may be termed *possible neuropathic pain*.

### 4.2. Probable neuropathic pain

The next level of certainty requires supporting evidence obtained by a clinical examination. The examination should optimally confirm the presence of negative sensory signs, ie, partial or complete loss to one or several sensory modalities concordant with the lesion or disease of the somatosensory nervous system (eg, light touch, cold temperature, Tables 1 and 2).

**Table 2.**
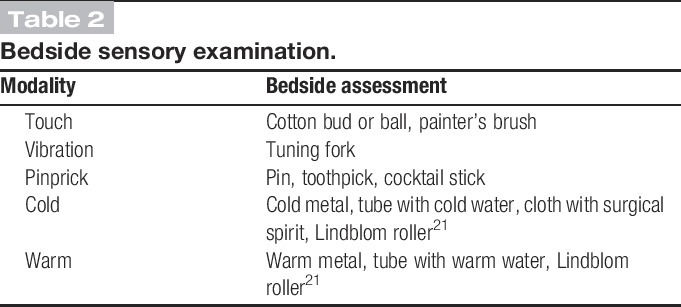
Bedside sensory examination.

Demonstrating sensory loss to one or more of these modalities and delineation of the area affected by the negative sensory phenomena are central to the determination as to whether a nervous system lesion is the cause of the sensory disturbance (ie, whether it is compatible with neuropathy). Negative sensory signs may also be seen in nociceptive pain, but in these cases they lack neuroanatomically distinct borders and are not reproducible.^12,19^ The sensory signs may or may not be accompanied by motor or autonomic signs.

Positive sensory signs alone (eg, pressure-evoked hyperalgesia) carry less weight towards neuropathic pain probability, in particular, if their distribution does not follow relevant neuroanatomical delineation. Positive sensory symptoms and signs may be seen in patients with other conditions such as inflammatory pain, pain of unknown origin, anxiety, and sleep deprivation, and can be affected by stress and negative emotions.^25,34^ It is important to emphasize that there are conditions where sensory loss is not a prerequisite for a neuropathic pain condition. In certain neuropathic pain conditions such as hereditary channelopathies^1,33^ and in subgroups of patients with, eg, peripheral nerve injury,^20^ touch-evoked allodynia or thermal hyperalgesia may be present without detectable sensory loss. The presence of such positive signs may mask sensory loss in some of these patients.

Idiopathic or classical trigeminal neuralgia is a special case. In trigeminal neuralgia, sensory deficits may not be found on clinical examination, although quantitative sensory testing may show sensory abnormality.^20^ In these cases, a history of characteristic triggering maneuvers may be counted as positive sensory signs. They can sometimes be repeated by the examiner, who may thus evoke and see the characteristic *tic*. Another special case is painful channelopathies as they are often paroxysmal and sensory examination can be normal between attacks. A history of characteristic symptoms may be considered a surrogate for positive sensory signs. In phantom pain, a sensory examination is not possible in the pain area. In these cases, the loss of the body part where pain is perceived is counted as a surrogate for sensory signs within the pain distribution.

Often, sensory changes to light touch, vibration, pinprick, cold, or warmth can be confirmed by a clinical examination (Table 2), but more detailed analysis using quantitative sensory testing may be needed.^13^ Prolonged pain after herpes zoster is associated with sensory abnormalities in a neuroanatomically plausible distribution in most, but not all cases.^20^ In rare cases where sensory abnormalities are doubtful or lacking, documentation of a herpes zoster rash in the form of a photograph or clinical record will add to the evidence of somatosensory pathway involvement, allowing a subsequent designation of the condition as probable neuropathic pain. Sensory function is difficult to evaluate in deep tissue and viscera. For that reason, a level of certainty beyond possible neuropathic pain can rarely be obtained for visceral or deep somatic types of pain. Innervation territories of nerves and roots vary between individuals, they are not always clearly demarcated, and there is often overlap between them. Because of central sensitization phenomena, the areas of allodynia and hyperalgesia may extend beyond the innervation territory.^35^

### 4.3. Definite neuropathic pain

The final level of certainty requires that an objective diagnostic test confirms the lesion or disease of the somatosensory nervous system. This may not always be possible in the nonspecialist environment. Examples of such diagnostic tests include computed tomography, magnetic resonance imaging, or other imaging techniques to confirm the presence of stroke, multiple sclerosis, spinal cord injury, or nerve lesion; skin biopsy showing reduced intraepidermal nerve fiber density, neurophysiological tests such as nerve conduction velocity, heat and laser evoked potentials, nerve excitability tests, R1 blink reflex demonstrating neural function compromise, microneurography with evidence of aberrant nociceptor activity; and genetic tests confirming a hereditary neuropathic pain disorder such as inherited erythromelalgia.^13^ In cases of amputation or a surgeon's clear verification of an intraoperative nerve lesion, further diagnostic tests are not necessary to arrive at the grading of “definite” neuropathic pain, because direct anatomical or surgical evidence counts as a confirmatory test.

This final level is reached by using only positive criteria for the location and nature of the neurological lesion or disease, without excluding other potential causes of the pain. Patients can have nociceptive pain in an area within the territory affected by an injury or disease involving the nervous system. Examples include spasticity-related pain below the level of injury in a patient with incomplete spinal cord injury, shoulder pain because of a lesion of the rotator cuff tendons in the area with sensory abnormalities after a stroke, inflammatory pain in the innervation territory of the lesioned nerves after thoracotomy or herniotomy, and plantar fasciitis in a patient with polyneuropathy. In these cases, despite fulfilling all 4 criteria of the grading system, the pain may still not be neuropathic. Thus, it is important to note that the final level does not completely rule out the possibility that other conditions such as tissue inflammation may fully or partially explain the pain. This remaining uncertainty about causality between the identified lesion or disease and the clinical presentation of the patient is a common situation in neurological diagnostics. In this context, the term definite neuropathic pain means that the clinician, by using history, clinical examination and auxiliary testing, is able to reach the level of confirming clinically that a patient has a neurological lesion that can explain the pain. Because the grading system only determines the level of certainty with which the presence or absence of a lesion or disease of the somatosensory nervous system *can* explain the pain, it is always important to consider if other causes for the patient's pain conditions may be present.

### 4.4. Summary

Compared to the grading system published in 2008, we have

(1) changed the order of the grading criteria to better reflect clinical practice.
(2) annotated the terms used to improve clarity.
(3) recognized the role of screening tools (questionnaires) in neuropathic pain evaluation.
(4) emphasized that reaching the final level of certainty (definite neuropathic pain) confirms clinically that a lesion or disease of the somatosensory nervous system can explain the pain but, as often in neurology, it does not establish causality (ie, there may still be other causes of the pain such as a diabetic ulcer).

The main purpose of the grading system is to help in the classification of the pain as neuropathic. Other types of pain include nociceptive pain, which is pain that arises from actual or threatened damage to non-neural tissue and is due to the activation of nociceptors,^16^ and pain that does not fulfill the criteria for either nociceptive or neuropathic pain, such as chronic widespread pain, fibromyalgia, and irritable bowel syndrome.^31^ Furthermore, patients may have 2 or more types of pain, and the classification of pain may be particularly difficult if more than one type of pain exist in the same area.

The grading system is intended for use in individual patients in the clinic and for research, but not for classification of diseases. Some patients diagnosed with fibromyalgia may have small fiber pathology and may fulfill the criteria for neuropathic pain whereas others may not.^9,26^ In addition, patients with complex regional pain syndrome (CRPS) type II fulfill the criteria for neuropathic pain, whereas patients with type I do not. Complex regional pain syndrome type I is by definition a condition in which no nerve lesion can be verified.^5^ Although an inflammatory reaction, considered to be at the core of development of CRPS, can conceivably damage nociceptors, the current pathophysiological evidence for it is limited and inconsistent, and thus does not justify the designation by default of CRPS type I as neuropathic pain.^7,23^ Similarly, individual patients with Parkinson's disease may have neuropathic pain if there is a documented lesion of the somatosensory system and they fulfill the grading criteria, but musculoskeletal pain is an important differential diagnosis. The demonstration of reduced small nerve fibers obtained from skin biopsies in patients with fibromyalgia and Parkinson's disease is at the moment not sufficient evidence per se for labeling pain in these patients as neuropathic. There is currently no evidence to indicate that patients with cluster headache or migraine have lesions of the somatosensory system.

## 5. Limitations and future directions

Based on the narrative literature review and discussions in the committee, we identified several weaknesses in our current knowledge about neuropathic pain and issues that need to be addressed in the future.

(1) The lack of positive criteria for identifying non-neuropathic pain, and the lack of pathognomonic features of neuropathic pain make it difficult to reach a level of “definite” neuropathic pain. Previous attempts to define a gold standard for neuropathic pain have been hampered by the inherent circular bias imposed by the fact that the criteria for defining clinical neuropathic pain are also used as measures in newly introduced tools. One important area of research will be to use the present grading system as a reference standard against which other neuropathic pain approaches should be systematically validated. In this goal, it will be important to perform field testing of this system, in particular, to assess its test-retest reliability and inter-rater reliability.
(2) The lack of pain diagnostic tools for low resource settings and the need for more educational efforts.^22^ Screening tools may be helpful, but at a single-patient level, they may wrongly classify some patients (false-positive) or fail to identify other patients (false-negative).^3,32^
(3) In research based only on clinical history, such as questionnaire surveys and patient interviews, only the level of “possible” neuropathic pain can be reached and even then only sometimes. Although validated questionnaires exist, these questionnaires were mostly developed based on the old and less precise IASP definition, and there is no validated approach to defining relevant pain distribution and history.^29^
(4) Variability of innervation territories of roots, nerves and fascicles as well as difficulty in quantitating sensory function in deep tissues such as joints and muscles and visceral tissues can make it difficult to identify neuropathic pain in certain cases, and current textbook figures are based on often imprecise renditions of very old data from relatively small case series. In future, it is important to generate probabilistic maps of innervation territories and to develop methods for the assessment of sensory function in joints and muscles and visceral tissues. Identification of appropriate control areas for specific conditions, establishment of inter- and intra-examiner reliability, and assessment of the use of patient self-examination^14,27^ are also needed.
(5) The possible presence of neuropathic pain in conditions where lesions or diseases of the somatosensory nervous system occur secondary to an inflammatory condition is not clear. Examples are osteoarthritis, in which there may be a decrease in the innervations in the synovial lining layer and increase in the innervations of cartilage with concomitant sensory abnormalities in the skin,^28^ and chronic pancreatitis, in which histopathology shows evidence of local nerve involvement.^8^

## 6. Conclusions

Neuropathic pain is a term used for a group of conditions with a wide range of causes and different pain distributions. However, all these conditions are characterized by a lesion or disease affecting the somatosensory nervous system peripherally or centrally. The grading system represents a tool to determine the level of certainty that the pain in an individual is neuropathic in nature. Such grading is naturally based on clinical judgment. Therefore, it relies heavily on the experience, skills, and resources available for assessment.

We anticipate that the rephrasing and reordering of the 4 criteria of the grading system will facilitate its use by both neurologists and non-neurologists. The level “probable” should usually be sufficient to initiate treatment according to neuropathic pain guidelines. The level “definite” is useful in specialist contexts and when a causal treatment of the underlying lesion or disease is an option. In some cases, sensory signs in the painful area may be difficult to demonstrate, although the nature of the lesion or disease is confirmed (eg, trigeminal neuralgia, channelopathies, postherpetic neuralgia); for these cases, the level “probable” continues to be appropriate if a diagnostic test confirms the lesion or disease of the somatosensory nervous system.

This paper includes an update of the grading system published in 2008.^30^ The goal of this update is to provide a revised grading system that is clinically useful, internally consistent, and allows appropriate treatment decisions in the face of uncertainty. We anticipate that the revised grading system will be incorporated into the “content model” of neuropathic pain in the upcoming 11th version of the International Classification of Diseases.^31^ We have also identified a range of important research topics that will further improve the classification and grading of neuropathic pain in the future.

## Conflict of interest statement

The authors have no conflicts of interest to declare.

R-D. Treede and T. S. Jensen contributed equally to this work.

Supported by the NeuPSIG of the International Association for the Study of Pain. S.N. Raja was partly supported by NIGMS, NS26363. D.L.H. Bennett is a Wellcome Senior Clinical Scientist (Ref 095698z/11/z). D. Bouhassira, D.L.H. Bennett, R. Baron, N.B. Finnerup, T.S. Jensen, A.S.C. Rice, and B.H. Smith are members of the DOLORisk consortium funded by the European Commission Horizon 2020 (ID633491). D.L.H. Bennett, N.B. Finnerup, and T.S. Jensen are members of the International Diabetic Neuropathy Consortium, the Novo Nordisk Foundation, grant number NNF14SA0006. R. Baron and R-D. Treede are members of the German Research Network on Neuropathic Pain (DFNS). R. Baron, D.L.H. Bennett, N.B. Finnerup, A.S.C. Rice, J. Serra, T.S. Jensen, and R-D. Treede are members of the IMI-Europain consortium. Ralf Baron has received grants/research support from Pfizer, Genzyme, Grünenthal, Mundipharma. He is a member of the EU Project No 633491: DOLOR-isk. A member of the IMI “Europain” collaboration and industry members of this are: Astra Zeneca, Pfizer, Esteve, UCB-Pharma, Sanofi Aventis, Grünenthal, Eli Lilly and Boehringer Ingelheim. German Federal Ministry of Education and Re-search (BMBF): Member of the ERA_NET NEU-RON/IM-PAIN Project. German Research Net-work on Neuropathic Pain, NoPain system biolo-gy. German Research Foundation (DFG). He has received speaking fees from Pfizer, Genzyme, Grünenthal, Mundipharma, Sanofi Pasteur, Medtronic, Eisai, Lilly, Boehringer Ingelheim, Astellas, Desitin, Teva Pharma, Bayer-Schering, MSD, bioCSL. He has been a consultant for Pfizer, Genzyme, Grünenthal, Mundipharma, Allergan, Sanofi Pasteur, Medtronic, Eisai, Lilly, Boehringer Ingelheim, Astellas, Novartis, Bristol-Myers-Squibb, Biogenidec, AstraZeneca, Merck, Abbvie, Daiichi Sankyo, Glenmark Pharmaceuticals, bioCSL an Teva Pharma.

## References

[1] Bennett DL Woods CG Painful and painless channelopathies. Lancet Neurol 2014;13:587–99. 24813307 10.1016/S1474-4422(14)70024-9

[2] Bennett M The LANSS Pain Scale: the Leeds assessment of neuropathic symptoms and signs. PAIN 2001;92:147–57. 11323136 10.1016/s0304-3959(00)00482-6

[3] Bennett MI Attal N Backonja MM Baron R Bouhassira D Freynhagen R Scholz J Tolle TR Wittchen HU Jensen TS Using screening tools to identify neuropathic pain. PAIN 2007;127:199–203. 17182186 10.1016/j.pain.2006.10.034

[4] Bennett MI Rayment C Hjermstad M Aass N Caraceni A Kaasa S Prevalence and aetiology of neuropathic pain in cancer patients: a systematic review. PAIN 2012;153:359–65. 22115921 10.1016/j.pain.2011.10.028

[5] Birklein F O'Neill D Schlereth T Complex regional pain syndrome: An optimistic perspective. Neurology 2015;84:89–96. 25471395 10.1212/WNL.0000000000001095

[6] Bouhassira D Attal N Alchaar H Boureau F Brochet B Bruxelle J Cunin G Fermanian J Ginies P Grun-Overdyking A Jafari-Schluep H Lanteri-Minet M Laurent B Mick G Serrie A Valade D Vicaut E Comparison of pain syndromes associated with nervous or somatic lesions and development of a new neuropathic pain diagnostic questionnaire (DN4). PAIN 2005;114:29–36. 15733628 10.1016/j.pain.2004.12.010

[7] Caty G Hu L Legrain V Plaghki L Mouraux A Psychophysical and electrophysiological evidence for nociceptive dysfunction in complex regional pain syndrome. PAIN 2013;154:2521–8. 23891894 10.1016/j.pain.2013.07.038

[8] Ceyhan GO Demir IE Maak M Friess H Fate of nerves in chronic pancreatitis: Neural remodeling and pancreatic neuropathy. Best Pract Res Clin Gastroenterol 2010;24:311–22. 20510831 10.1016/j.bpg.2010.03.001

[9] Doppler K Rittner HL Deckart M Sommer C Reduced dermal nerve fiber diameter in skin biopsies of patients with fibromyalgia. PAIN 2015;156:2319–25. 26164586 10.1097/j.pain.0000000000000285

[10] Finnerup NB Attal N Haroutounian S McNicol E Baron R Dworkin RH Gilron I Haanpaa M Hansson P Jensen TS Kamerman PR Lund K Moore A Raja SN Rice AS Rowbotham M Sena E Siddall P Smith BH Wallace M Pharmacotherapy for neuropathic pain in adults: a systematic review and meta-analysis. Lancet Neurol 2015;14:162–73. 25575710 10.1016/S1474-4422(14)70251-0 PMC4493167

[11] Freynhagen R Baron R Gockel U Tölle TR painDETECT: a new screening questionnaire to identify neuropathic components in patients with back pain. Curr Med Res Opin 2006;22:1911–20. 17022849 10.1185/030079906X132488

[12] Geber C Magerl W Fondel R Fechir M Rolke R Vogt T Treede RD Birklein F Numbness in clinical and experimental pain–a cross-sectional study exploring the mechanisms of reduced tactile function. PAIN 2008;139:73–81. 18423989 10.1016/j.pain.2008.03.006

[13] Haanpaa M Attal N Backonja M Baron R Bennett M Bouhassira D Cruccu G Hansson P Haythornthwaite JA Iannetti GD Jensen TS Kauppila T Nurmikko TJ Rice AS Rowbotham M Serra J Sommer C Smith BH Treede RD NeuPSIG guidelines on neuropathic pain assessment. PAIN 2011;152:14–27. 20851519 10.1016/j.pain.2010.07.031

[14] Hoimyr H Rokkones KA von Sperling ML Finnerup K Jensen TS Finnerup NB Persistent pain after lymph node excision in patients with malignant melanoma is neuropathic. PAIN 2011;152:2721–8. 21871733 10.1016/j.pain.2011.07.009

[15] IASP. Global year against neuropathic pain. 2015; Available at: http://www.iasp-pain.org/GlobalYear/NeuropathicPain?navItemNumber=580. Accessed September 24, 2015.

[16] IASP. ISAP taxonomy. 2012; Available at: http://www.iasp-pain.org/Education/Content.aspx?ItemNumber=1698&navItemNumber=576. Accessed September 24, 2015.

[17] Jensen TS Baron R Haanpaa M Kalso E Loeser JD Rice AS Treede RD A new definition of neuropathic pain. PAIN 2011;152:2204–5. 21764514 10.1016/j.pain.2011.06.017

[18] Krause SJ Backonja MM Development of a neuropathic pain questionnaire. Clin J Pain 2003;19:306–14. 12966256 10.1097/00002508-200309000-00004

[19] Leffler AS Hansson P Kosek E Somatosensory perception in patients suffering from long-term trapezius myalgia at the site overlying the most painful part of the muscle and in an area of pain referral. Eur J Pain 2003;7:267–76. 12725850 10.1016/S1090-3801(02)00138-6

[20] Maier C Baron R Tolle TR Binder A Birbaumer N Birklein F Gierthmuhlen J Flor H Geber C Huge V Krumova EK Landwehrmeyer GB Magerl W Maihofner C Richter H Rolke R Scherens A Schwarz A Sommer C Tronnier V Uceyler N Valet M Wasner G Treede RD Quantitative sensory testing in the German Research Network on Neuropathic Pain (DFNS): somatosensory abnormalities in 1236 patients with different neuropathic pain syndromes. PAIN 2010;150:439–50. 20627413 10.1016/j.pain.2010.05.002

[21] Marchettini P Marangoni C Lacerenza M Formaglio F The Lindblom roller. Eur J Pain 2003;7:359–64. 12821407 10.1016/S1090-3801(03)00052-1

[22] Mick G Baron R Correa-Illanes G Hans G Mayoral V Frias X Sintes D Keller T Is an easy and reliable diagnosis of localized neuropathic pain (LNP) possible in general practice? Development of a screening tool based on IASP criteria. Curr Med Res Opin 2014;30:1357–66. 24650347 10.1185/03007995.2014.907562

[23] Oaklander AL Fields HL Is reflex sympathetic dystrophy/complex regional pain syndrome type I a small-fiber neuropathy? Ann Neurol 2009;65:629–38. 19557864 10.1002/ana.21692

[24] Portenoy R Development and testing of a neuropathic pain screening questionnaire: ID Pain. Curr Med Res Opin 2006;22:1555–65. 16870080 10.1185/030079906X115702

[25] Schuh-Hofer S Wodarski R Pfau DB Caspani O Magerl W Kennedy JD Treede RD One night of total sleep deprivation promotes a state of generalized hyperalgesia: a surrogate pain model to study the relationship of insomnia and pain. PAIN 2013;154:1613–21. 23707287 10.1016/j.pain.2013.04.046

[26] Serra J Collado A Sola R Antonelli F Torres X Salgueiro M Quiles C Bostock H Hyperexcitable C nociceptors in fibromyalgia. Ann Neurol 2014;75:196–208. 24243538 10.1002/ana.24065

[27] Sorensen BS Jorgensen J Jensen TS Finnerup NB Pain following blood donation: a questionnaire study of long-term morbidity (LTM) in blood donors. Vox Sang 2015;109:18–24. 25827316 10.1111/vox.12245

[28] Thakur M Dickenson AH Baron R Osteoarthritis pain: nociceptive or neuropathic? Nat Rev Rheumatol 2014;10:374–80. 24686507 10.1038/nrrheum.2014.47

[29] Torrance N Ferguson JA Afolabi E Bennett MI Serpell MG Dunn KM Smith BH Neuropathic pain in the community: more under-treated than refractory? PAIN 2013;154:690–9. 23485369 10.1016/j.pain.2012.12.022 PMC3630326

[30] Treede RD Jensen TS Campbell JN Cruccu G Dostrovsky JO Griffin JW Hansson P Hughes R Nurmikko T Serra J Neuropathic pain: redefinition and a grading system for clinical and research purposes. Neurology 2008;70:1630–5. 18003941 10.1212/01.wnl.0000282763.29778.59

[31] Treede RD Rief W Barke A Aziz Q Bennett MI Benoliel R Cohen M Evers S Finnerup NB First MB Giamberardino MA Kaasa S Kosek E Lavand'homme P Nicholas M Perrot S Scholz J Schug S Smith BH Svensson P Vlaeyen JW Wang SJ A classification of chronic pain for ICD-11. PAIN 2015;156:1003–7. 25844555 10.1097/j.pain.0000000000000160 PMC4450869

[32] Vaegter HB Andersen PG Madsen MF Handberg G Enggaard TP Prevalence of neuropathic pain according to the IASP grading system in patients with chronic non-malignant pain. Pain Med 2014;15:120–7. 24165161 10.1111/pme.12273

[33] Waxman SG Merkies IS Gerrits MM Dib-Hajj SD Lauria G Cox JJ Wood JN Woods CG Drenth JP Faber CG Sodium channel genes in pain-related disorders: phenotype-genotype associations and recommendations for clinical use. Lancet Neurol 2014;13:1152–60. 25316021 10.1016/S1474-4422(14)70150-4

[34] Wiech K Tracey I The influence of negative emotions on pain: behavioral effects and neural mechanisms. Neuroimage 2009;47:987–94. 19481610 10.1016/j.neuroimage.2009.05.059

[35] Woolf CJ Central sensitization: implications for the diagnosis and treatment of pain. PAIN 2011;152:S2–15. 20961685 10.1016/j.pain.2010.09.030 PMC3268359

